# The effect of date fruit consumption on early postpartum hemorrhage: a randomized clinical trial

**DOI:** 10.1186/s12905-023-02604-9

**Published:** 2023-08-23

**Authors:** Maryam Niknami, Maryam Farash, Mona Rahnavardi, Saman Maroufizadeh, Roya Faraji Darkhaneh

**Affiliations:** 1 Department of Midwifery, School of Nursing and Midwifery, Guilan University of Medical Sciences, Rasht, Iran; 2 MSc Student of Midwifery, School of Nursing and Midwifery, Guilan University of Medical Sciences, Rasht, Iran; 3 Department of Biostatistics and Epidemiology, School of Health, Guilan University of Medical Sciences, Rasht, Iran; 4 Professor of Obstetrics & Gynecology, Reproductive Health Research Center, Department of Obstetrics & Gynecology, Al-Zahra Hospital, School of Medicine, Guilan University of Medical Sciences, Rasht, Iran

**Keywords:** Postpartum hemorrhage, Phoeniceae, Date fruit, Delivery, Obstetric

## Abstract

**Background:**

Postpartum hemorrhage, anemia, and iron deficiency are important health problems. Using safe, cheap, and available methods to reduce the amount of hemorrhage after childbirth can be effective for the mother's health during this period. Therefore, this study was conducted to determine the effect of date fruit consumption on the amount of hemorrhage after natural childbirth.

**Methods:**

This randomized clinical trial was conducted on 98 women referred to the maternity ward of Al-Zahra Hospital in Rasht using the available sampling method. The primary outcome was postpartum hemorrhage, measured using the Pictorial Blood Loss Assessment Chart (PBLAC). Two hours after delivery, 100 g of date fruits were given to the intervention group, and the amount of hemorrhage was recorded during the first 24 h. Comparison between the two groups was done with the Mann–Whitney test with the Hodges–Lehmann estimator and corresponding exact conditional nonparametric confidence interval (CI) as effect estimate. A *P* < 0.05 was considered significant.

**Results:**

The median of postpartum hemorrhage after normal delivery in the date and control groups was 35.0 [interquartile range (IQR): 22.0 to 39.8] and 39.0 [IQR: 27.5 to 64.5], respectively. Using the Hodges–Lehmann estimator, on average, the median postpartum hemorrhage in the date group was 9.0 (95% CI: 2.00–18.0) units lower than the control group (*P* = 0.009).

**Conclusion:**

Consumption of dates effectively reduces the amount of hemorrhage after natural childbirth; thus, consuming this fruit during postpartum period is recommended. Also to confirm the findings, it is recommended to conduct similar studies in this field.

**Trial registration:**

This trial was registered with the Iranian Registry of Clinical Trials; https://www.irct.ir/trial/59197 (IRCT20210607051505N2) on 31/10/2021.

## Background

Hemorrhage (500 ccs or more) during the first 24 h after a normal delivery is defined as a primary postpartum hemorrhage [1]. The global prevalence of postpartum hemorrhage is 5% of all births [2]. In Iran, the prevalence of hemorrhage has been estimated at 33.3% [3]. In developing countries, women are more exposed to issues, such as improper nutrition and anemia; hence, postpartum hemorrhage of 500 ccs can cause significant complications [4]. Fatigue, depression, anxiety, hysterectomy, hospitalization in the intensive care unit, and maternal death are some complications of postpartum hemorrhage [5]. Also, postpartum hemorrhage is one of the main causes of postpartum anemia [6]. Anemia in the postpartum period increases the prevalence of fatigue, shortness of breath, and heart palpitations, decreases cognitive ability, increases the risk of postpartum depression, and increases the risk of infections [7]. Therefore, diagnosing and treating postpartum hemorrhage is necessary to reduce maternal complications [8].

Most cases of postpartum hemorrhage can be controlled by the preventive use of uterotonics during the third stage of labor [2]. Oxytocin is the most commonly used uterotonic [9]. On the other hand, today, herbal medicines are also considered to prevent hemorrhage. In this regard, the effect of plants, such as Capsella Bursa Pastoris, chamomile, grape seed powder, cumin oil, Plantago, Urtica Dioica, and dates on postpartum hemorrhage has been widely studied [10–18].

Dates have powerful health benefits and are used in countries like Iran, India, Egypt, Morocco, and Iraq [19]. Dates are a rich source of micronutrients and macronutrients, and per 100 g of dates, contain 64.2 mg of magnesium, 0.5 mg of zinc, 6.03 mg of iron, 70.7 mg of calcium, 864 mg of potassium, and 10.5 µg of vitamin A. This fruit contains serotonin, and natural antioxidants, such as phenolic acid, flavonoids, and tannin [20, 21]. Due to its astringent properties, tannin causes contraction in the muscles of the uterus and myometrium [18, 22].

Although the effect of dates on postpartum hemorrhage has been studied, the results are contradictory. Khadem et al. compared the amount of hemorrhage in the first, second, and third hours after delivery in two groups of data and oxytocin recipients. The amount of hemorrhage in the first hour after delivery was significantly lower in the date group than in the oxytocin group. However, this difference was not statistically significant in the second and third hours after delivery [22]. Mojahed et al. investigated the effect of dates on hemorrhage in the first two hours after delivery. They showed that the average hemorrhage at the end of the first two hours after delivery in the oxytocin and date group was significantly lower than in the oxytocin group [10]. Also, Yadegari et al. assessed the effect of dates on the amount and duration of postpartum hemorrhage. The amount of hemorrhage on the first day in the intervention group was lower than in the control group, but this difference was not statistically significant [18]. However, Izzaddin and Razali investigated the effects of dates on labor and vaginal delivery and found no significant relationship between the consumption of dates and the amount of maternal hemorrhage [23, 24].

There is not enough information on the effect of dates on the amount of postpartum bleeding. On the other hand, considering the wide range of symptoms and complications caused by postpartum hemorrhage, using safe and available methods to reduce this condition can effectively improve mothers’ health in the postpartum period [18]. Therefore, the present study was conducted to determine the effect of date consumption on postpartum hemorrhage after a natural delivery.

## Methods

This study was a two-group non-blinded randomized clinical trial conducted on 98 women referred to the maternity ward of Al-Zahra Hospital, Rasht, in 2021. The protocol of the trial was approved by the Ethics Committee of Guilan University of Medical Sciences (code: IR.GUMS.REC.1400.309) and registered at the Iranian Clinical Trial Registration Center (code: IRCT20210607051505N2). Then, a letter of introduction was presented to the head of Al-Zahra Hospital, Rasht, and sampling was done. In addition to obtaining informed consent, the participants were informed about the study's objectives, its method, and the confidentiality of the information. Free pads in the same size and brand were provided to all participants. They were also assured that at the end of 24 h, they would be informed of the normal or abnormal amount of hemorrhage. The corresponding nurse and ward manager will be informed if the excessive hemorrhage is detected. The study lasted from November 2021 to May 2022.

### Sample size estimation

The sample size was estimated to be 39 people in each group (total sample size: 78 people) according to Yadegari's study [18] and considering α = 0.05, β = 0.2, the effect size d = 0.65 (relatively large), and the hemorrhage rate and the researcher's expectation of practical (clinical) difference, which was finally reduced to 49 cases per group (final total sample size: 98 people) because of the possible attrition of 20%.

$$\mathrm n\;=\;\frac{2\left({\mathrm Z}_{1-\mathrm a/2}\;+\;{\mathrm Z}_{1-\mathrm\beta}\right)^2}{\mathrm d^2}\;+\;\frac{\mathrm Z_{1-\mathrm a/2}^2}4$$

$$\alpha\;=\;0.05,\;Z_{1-\alpha/2}\;=Z_{0.975}\;=\;1.960$$

$$\begin{array}{cc}\beta =0.2,& {Z}_{1-\beta }\end{array}={Z}_{0.8}=0.841$$

$$\mathrm n\;=\;\frac{2\left(Z_{1-\alpha/2}\;+\;Z_{1-\beta}\right)^2}{d^2}\;+\;\frac{Z_{1-\alpha/2}^2}4\;=\;\frac{2\left(1.960\;+\;0.841\right)^2}{0.65^2}\;+\;\frac{\left(1.960\right)^2}4\;\cong39$$

### Inclusion and exclusion criteria

Inclusion criteria were mother's age between 18 and 35 years, at least primary education of the mother, history of giving birth less than five times, mother's body mass index between 18.5 and 29.9 kg/m^2^, no history of medical diseases and pregnancy complications (obesity, diabetes, preeclampsia, high blood pressure, anemia, coagulation disorders, placental disorders, hemorrhage during pregnancy, receiving hormonal drugs during pregnancy, and history of postpartum hemorrhage), not receiving herbal treatments during pregnancy, fetal estimated weight between 2500 and 4500 g, term pregnancy (37 to 42 weeks) and live birth, singleton pregnancy, vertex presentation, normal amniotic fluid index based on third trimester ultrasound, normal length of the first, second, and third stages based on partograph, no rupture of fetal membranes for more than 12 h, vaginal delivery without using vacuum or forceps, no precipitous delivery, no third- or fourth-degree rupture, no placental retention, no placental discharge with courage, no use of oxytocin more than the hospital's routine dose, no severe bleeding in the fourth stage of labor, and breastfeeding less than two hours after delivery. Exclusion criteria were no willingness to participate in the study, failure to complete the pictorial blood chart form, and the need for blood-reducing drugs (ergometrine, methylergonovine, and prostaglandins) during the first 24 h after delivery, eating less than 50 g of dates in the intervention group, date consumption in the control group, and consumption of other herbal medicines during the first 24 h by the intervention and control groups.

### Randomization

The subjects were selected using the available sampling method. The eligible subjects were divided into the date and control groups using the block randomization method with four and six block sizes. The Sealed Envelope Simple Randomization Service (2019) was used to generate the randomization list, and sequentially numbered; opaque sealed envelopes (SNOSE) were used to hide the allocation of participants.

### Study design

The intervention group received 100 g of Mazafati Bam dates two hours after delivery. The data collection tool was a questionnaire on personal and fertility information and the Pictorial Blood Loss Assessment Chart (PBLAC). The face validity method was used to check the validity of the personal information and fertility questionnaire. The PBLAC was first introduced by Higham in 1990 in the UK [25]. It is a standard tool, and its validity and reliability have been proven in several studies, such as those by Bokaei et al., Abedian et al., and Yadegari et al., and it has been introduced as a valid and reliable tool [18, 26, 27]. In the present study, it was used to record hemorrhage in the first 24 h after delivery. This tool includes a table in which the row shows the days of hemorrhage, and the columns represent lightly, moderately, and heavily soaked pads and blood clots according to size. A factor of one is given for a mild degree, a factor of five for a moderate degree, a factor of 20 for the pad being completely covered with blood, a score of one is considered for small clots, and a score of five for a large clot.

After each change, the subjects mark the corresponding place in the chart according to the amount of blood observed on the pad. At the end of the first 24 h after delivery, each mark is multiplied by the corresponding coefficient, and the obtained numbers are added to calculate the total score. A score of 100 and more indicates severe bleeding [25, 28]. The participants in both groups were also provided with pads to have a uniform hemorrhage record. Also, the PBLAC was provided to both groups, and they were asked to complete the chart during the first 24 h after delivery after changing the pads. Necessary information about how to complete the chart was provided to the subjects. At the end of the first 24 h after delivery, the PBLAC was collected by the researcher.

### Statistical methods

In this study, continuous variables are presented as mean ± standard deviation (SD) and median [interquartile range (IQR)] and categorical variables as frequency (percentage). The distribution of postpartum hemorrhage score was assessed with Kolmogorov–Smirnov and Shapiro–Wilk tests. Because of the non-normal distribution of the data, a comparison between the two groups was done with the Mann–Whitney test with the Hodges–Lehmann estimator and corresponding exact conditional nonparametric confidence interval (CI) as effect estimate. Data analysis was done with SPSS for Windows, version 16.0 (SPSS Inc., Chicago, IL, USA) and MedCalc version 19.5.3 (MedCalc Software, Ostend, Belgium). A *P* < 0.05 was considered statistically significant.

## Results

### Participant’s characteristics

**Fig. 1:**
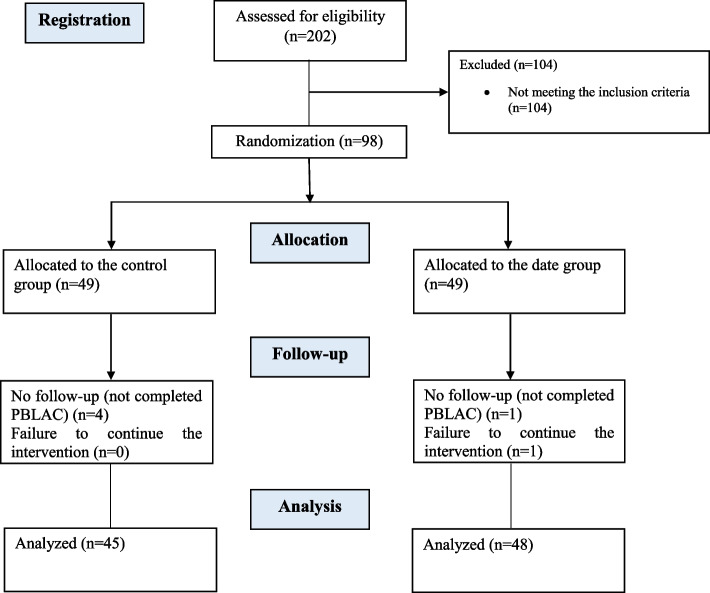
CONSORT flowchart of study participants

The flow of participants through the trial is in Fig. 1. A total of 202 women were screened, and 98 women underwent randomization. The first women underwent randomization on November 25, 2021, and the last on May 17, 2022. Of these, follow-up data were available for 93 mothers (48 mothers in the date group and 45 mothers in the control group) to be included in the modified intention-to-treat (mITT) analysis (Fig. 1). The mothers’ average age was 27.1 ± 5.5 years, and their average gestational age was 38.9 ± 1.1 weeks. The majority of mothers were primiparous (46.2%), and 38.7% were nulliparous; episiotomy was observed in 89 cases (95.7%), and 12 cases had participated in childbirth preparation classes (12.9%). All mothers experienced breastfeeding in the first two hours; the duration of breastfeeding in the first 2 h after the delivery was more than 10 min in 47 mothers (50.5%), and the frequency of breastfeeding in the first 24 h after the delivery was more than eight times in 40 mothers (43.0%). The average weight of babies was 3269.8 ± 438.3 g. The mean hemoglobin levels of mothers were 12.7 ± 0.1 g/dl (*P* > 0.05) (Table 1). Demographics and clinical characteristics were well-balanced between the control and date groups.

**Table 1:** Baseline characteristics of the women

	Total (*n* = 93)	Control (*n* = 45)	Date Consumption (*n* = 48)
Age (years)	27.1 ± 5.5	27.4 ± 5.7	26.8 ± 5.3
Weight (kg)	66.1 ± 11.0	66.4 ± 10.4	65.7 ± 11.6
Height (cm)	162.5 ± 6.9	162.9 ± 6.7	162.1 ± 7.0
BMI (kg/m2)	25.1 ± 3.6	25.0 ± 3.5	25.2 ± 3.8
Gestational age (weeks)	38.9 ± 1.1	38.8 ± 1.1	39.0 ± 1.1
Maternal hemoglobin levels (mg/dl)	12.7 ± 1.0	12.7 ± 1.0	12.6 ± 0.9
Duration of active phase (Hour)	3.6 ± 1.1	3.9 ± 1.2	3.4 ± 1.1
Second stage of labour (min)	19.1 ± 8.2	20.7 ± 8.6	17.6 ± 7.6
Third stage of labour (min)	5.1 ± 0.7	5.1 ± 0.7	5.1 ± 0.7
Weight of newborn (g)	3269.8 ± 438.3	3274.0 ± 447.6	3265.9 ± 434.1
	Frequency (Percentage)	Frequency (Percentage)	Frequency (Percentage)
Gravidity			
Primigravida	31 (33.3)	19 (42.2)	12 (25.0)
Gravid 2	39 (41.9)	15 (33.3)	24 (50.0)
Gravid 3 or more	23 (24.7)	11 (24.4)	12 (25.0)
Parity			
Nulliparous	36 (38.7)	20 (44.4)	16 (33.3)
Primiparous	43 (46.2)	19 (42.2)	24 (50.0)
Parity 2 or more	14 (15.1)	6 (13.3)	8 (16.7)
Participating in preparation classes for childbirth			
Yes	12 (12.9)	6 (13.3)	6 (12.5)
No	81 (87.1)	39 (86.7)	42 (87.5)
Episiotomy			
Yes	89 (95.7)	43 (95.8)	46 (95.8)
No	4 (4.3)	2(4.4)	2 (4.2)
Gender of newborn			
Male	46 (49.5)	26 (57.8)	20 (41.7)
Female	47 (50.5)	19 (42.2)	28 (58.3)

### Normality assumption

As presented in Table 2, both Kolmogorov–Smirnov test and Shapiro–Wilk test indicated that normality assumption was not met for postpartum hemorrhage score in both groups, therefore, Mann–Whitney test was used to evaluate the difference between groups.

**Table 2:** Evaluating the assumption of normality for postpartum hemorrhage score

	Control		Date Consumption	
	Statistic	*P*	Statistic	*P*
Kolmogorov–Smirnov test	0.198	< 0.001	0.166	< 0.001
Shapiro–Wilk test	0.852	< 0.001	0.897	0.001

### Effect of date consumption on postpartum hemorrhage

**Fig. 2:**
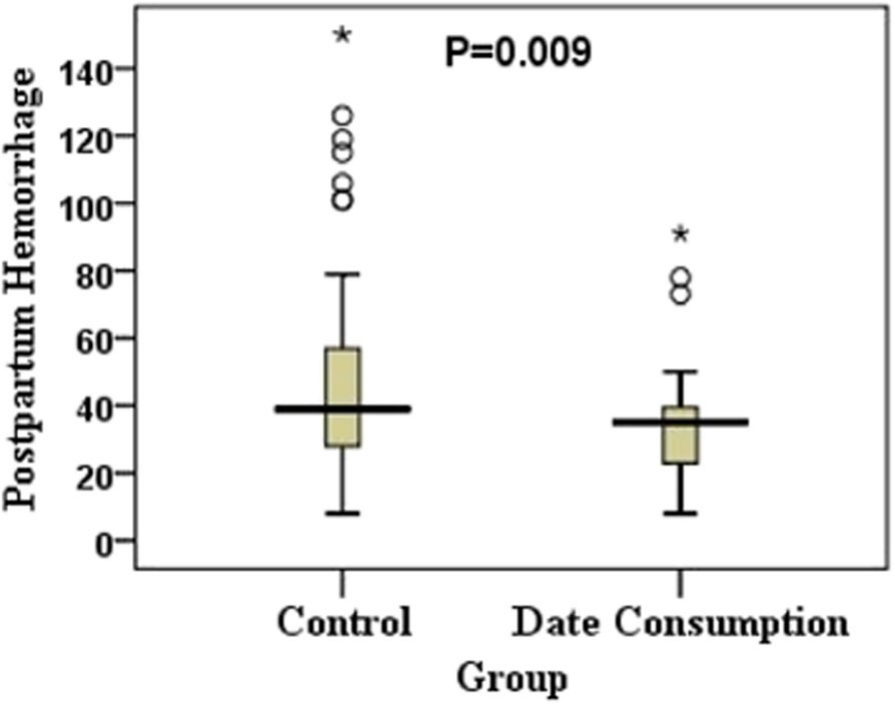
Comparison of postpartum hemorrhage scores after normal delivery between the date and control groups (using a box plot). Note. Box plot shows minimum, first quartile (Q1), median, third quartile (Q3), and maximum values. The outliers and extreme values are shown by the circle and asterisk, respectively. The *P* value is based on the Mann–Whitney test

The Mann–Whitney test showed that the postpartum hemorrhage in the date group was significantly lower than in the control group (35.0, IQR: 22.0 to 39.8 versus 39.0, IQR: 27.5 to 64.5; *P* = 0.009). Using the Hodges–Lehmann estimator, on average, the median score of postpartum hemorrhage in the date group were 9.0 (95% CI: 2.00–18.0) units lower than the control group (Table 3 and Fig. 2).

**Table 3:** Comparison of postpartum hemorrhage in the date and control groups

Group	Mean ± SD	Median [IQR]	Median Difference (95% CI)^a^	*P*
Control	50.9 ± 33.3	39.0 [27.5 to 64.5]	9.0 (2.0 to 18.0)	0.009
Date consumption	33.7 ± 16.7	35.0 [22.0 to 39.8]		

The *P* value is based on the Mann–Whitney test

*SD* Standard Deviation, *IQR* Interquartile Range, *CI* Confidence Interval

^a^ The median difference and 95% CI were calculated using the Hodges-Lehmann estimator

## Discussion

The current study was a randomized clinical trial to determine the effect of date consumption on the amount of hemorrhage after natural childbirth. The results showed that the amount of hemorrhage in the first 24 h after delivery in the date group was significantly lower than in the control group (P = 0.009). Yadegari et al. investigated the effect of dates on the amount and duration of postpartum hemorrhage. The amount of hemorrhage on the first day was less in the intervention group, but it was not statistically significant. Still, there was a statistically significant difference between the two groups regarding the amount of hemorrhage on the second to tenth days. Also, dates did not affect reducing the number of hemorrhage days [18]. Their results are consistent with our results. Khadem et al. compared the amount of hemorrhage in the first, second, and third hours after delivery in two date and oxytocin groups. In the first hour after delivery, the amount of hemorrhage in the date group was significantly lower than in the oxytocin group. Still, this difference was not statistically significant in the second and third hours after delivery. The total amount of hemorrhage in three hours was significantly lower in the date group than in the control group [22]. Mojahed et al. assessed the effect of date consumption on postpartum hemorrhage. The average hemorrhage at the end of the first 2 h after delivery in the oxytocin and date group was significantly lower than in the oxytocin group [10]. The studies above showed a decrease in the amount of hemorrhage after natural childbirth, which is consistent with the results of the present study.

On the other hand, Izzaddin et al. investigated the effects of date fruit consumption on labor and vaginal delivery in pregnant women. Date consumption decreased the duration of the first and third stages of labor. Still, it had no significant effect on other maternal outcomes, including the approximate amount of hemorrhage, which is inconsistent with the present research [23]. Also, Razali et al. investigated the effect of date consumption on labor and delivery outcomes. They showed no statistically significant difference in maternal hemorrhage in the intervention and control groups [24]. The results of these studies are not in line with the results of the present study, which can be related to the difference in the time of the intervention because in the present study, the time of the intervention was 2 h after delivery, but in these studies, the intervention was at the beginning of labor or earlier. Also, the difference in the results of the studies can be related to the demographic differences of the samples and the measurement tools, which can affect the result.

The limitations of the present study were using self-reporting tools to determine the amount of postpartum hemorrhage. It is suggested to carry out studies using interventions before delivery or continuing the intervention for ten days or more postpartum. One of the present study's strengths was eliminating many confounding variables, such as pregnancy complications, receiving herbal treatments, fetal estimated weight over the 4500 g, abnormal amniotic fluid index, severe bleeding, etc., from the inclusion criteria.

## Conclusion

According to the results of the present study, it can be concluded that the consumption of date after natural delivery led to a decrease in hemorrhage during the first 24 h after delivery. Therefore, the delivery and post-delivery ward officials and personnel can advise mothers to consume dates. On the other hand, healthcare managers can provide the necessary arrangements to include dates in the diet of mothers hospitalized in the postpartum ward of hospitals.

## Data Availability

Data from this study will be available upon request of the corresponding author.

## Abbreviations

PBLAC: Pictorial Blood loss Assessment Chart
SNOSE: Sequentially numbered, opaque sealed envelopes
mITT: Modified intention-to-treat

## References

[1] Kebede BA, Abdo RA, Anshebo AA, Gebremariam BM (2019). Prevalence and predictors of primary postpartum hemorrhage: An implication for designing effective intervention at selected hospitals, Southern Ethiopia. PLoS ONE.

[2] WHO Guidelines Approved by the Guidelines Review Committee. WHO recommendations: Uterotonics for the prevention of postpartum haemorrhage. Geneva: World Health Organization; 2018. 30645062

[3] Ashouri N, Kordi M, Shakeri M-T, Tara F (2019). Vaginal delivery postpartum hemorrhage: incidence, risk factors, and causes. Iranian J Obstet Gynecol Infertility.

[4] Begley CM, Gyte GM, Devane D, McGuire W, Weeks A, Biesty LM (2019). Active versus expectant management for women in the third stage of labour. Cochrane Database Syst Rev.

[5] Latt SM, Alderdice F, Elkington M, AwngShar M, Kurinczuk JJ, Rowe R (2023). Primary postpartum haemorrhage and longer-term physical, psychological, and psychosocial health outcomes for women and their partners in high income countries: A mixed-methods systematic review. PLoS ONE.

[6] Butwick AJ, Walsh EM, Kuzniewicz M, Li SX, Escobar GJ (2017). Patterns and predictors of severe postpartum anemia after Cesarean section. Transfusion.

[7] Wemakor A, Ziyaaba A, Yiripuo F (2022). Risk factors of anaemia among postpartum women in Bolgatanga Municipality. Ghana BMC nutrition.

[8] Borovac-Pinheiro A, Pacagnella RC, Cecatti JG, Miller S, El Ayadi AM, Souza JP (2018). Postpartum hemorrhage: new insights for definition and diagnosis. Am J Obstet Gynecol.

[9] Gallos ID, Papadopoulou A, Man R, Athanasopoulos N, Tobias A, Price MJ, et al. Uterotonic agents for preventing postpartum haemorrhage: a network meta‐analysis. Cochrane Database of Systematic Reviews. 2018(12) 10.1002/14651858.CD011689.pub3. 10.1002/14651858.CD011689.pub3 PMC6388086 30569545

[10] Mojahed S, Aflatunian A, Khadem N, DehghaniFirouzabadi R, Karimi Zarchi M (2012). An investigation into effectiveness of date (Rutab) on postpartum hemorrhage. SSU_J.

[11] Abedian Z, RezvaniFard M, Asili J, Esmaeili H, Dadgar S (2016). Comparison of the Effect of chamomile matricaria and mefenamic acid capsules on postpartum hemorrhage in women with postpartum pain. The Iranian J Obstet Gynecol Infertility.

[12] Fazel N, Esmaeili H, ShamaeianRazavi N (2013). Effect of cumin oil on post partum hemorrhage after cesarean. Iranian J Med Aromatic Plants Res.

[13] Ghalandari S, Kariman N, Sheikhan Z, Mojab F, Mirzaei M, Shahrahmani H (2017). Effect of hydroalcoholic extract of capsella bursa pastoris on early postpartum hemorrhage: a clinical trial study. J Altern Complement Med.

[14] Ghalandari S, Kariman N, Sheikhan Z, Shahrahmani H, Asadi N (2016). Systematic review on variety of effective treatment methods for postpartum hemorrhage in Iran and world. The Iranian J Obstet Gynecol Infertility.

[15] Izadpanah A, Alahyari E, Torshizi M, Khazaie Z, Sharifzadeh G, Hosseini M (2018). Effect of grape seed powder on postpartum hemorrhage in vaginal delivery: a randomized controlled clinical trial. The Iranian J Obstet Gynecol Infertility.

[16] Khojastehfard Z, Golmakani N, Mazloum SR, Hamedi SS, Feyzabadi Z, Mirteimouri M (2019). The effect of plantago major rectal suppository on postpartum hemorrhage rate in women at the risk of bleeding: a single-blind clinical trial. The Iranian J Obstet Gynecol Infertility.

[17] Naafe M, Kariman N, Keshavarz Z, Mojab F, Chaibakhsh S (2016). Considering the effect of hydro alcoholic extract of capsella bursa pastoris on menorrhagia. J Arak Uni Med Sci.

[18] Yadegari Z, Amir Ali Akbari S, Sheikhan Z, Nasiri M, Akhlaghi F (2016). The effect of consumption of the date fruit on the amount and duration of the postpartum bleeding. The Iranian J Obstet Gynecol Infertility.

[19] Echegaray N, Pateiro M, Gullon B, Amarowicz R, Misihairabgwi JM, Lorenzo JM (2020). Phoenix dactylifera products in human health–a review. Trends Food Sci Technol.

[20] Maqsood S, Adiamo O, Ahmad M, Mudgil P (2020). Bioactive compounds from date fruit and seed as potential nutraceutical and functional food ingredients. Food Chem.

[21] Qadir A, Shakeel F, Ali A, Faiyazuddin M (2020). Phytotherapeutic potential and pharmaceutical impact of Phoenix dactylifera (date palm): current research and future prospects. J Food Sci Technol.

[22] Khadem N, Sharaphy A, Latifnejad R, Hammod N, Ibrahimzadeh S (2007). Comparing the efficacy of dates and oxytocin in the management of postpartum hemorrhage. Shiraz E-Medical Journal.

[23] Ahmed IE, Mirghani HO, Mesaik MA, Ibrahim YM, Amin TQ (2018). Effects of date fruit consumption on labour and vaginal delivery in Tabuk, KSA. J Taibah University Med Sci.

[24] Razali N, MohdNahwari SH, Sulaiman S, Hassan J (2017). Date fruit consumption at term: effect on length of gestation, labour and delivery. J Obstet Gynaecol.

[25] Higham JM, O'Brien PM, Shaw RW (1990). Assessment of menstrual blood loss using a pictorial chart. Br J Obstet Gynaecol.

[26] Bokaie M, Enjezab B (2017). The effects of oral fennel extract on the intensity of menstrual bleeding in relieving dysmenorrheal: a randomized clinical trial. Commun Health J.

[27] Mirlashari BM, Abedian Z, Rakhshandeh H, Esmaily H (2019). Comparison of the effects of bromelain and mefenamic acid on menstrual bleeding in students with primary dysmenorrhea: a double-blind randomized clinical trial. The Iranian J Obstet Gynecol Infertility.

[28] Magnay JL, O’Brien S, Gerlinger C, Seitz C (2020). Pictorial methods to assess heavy menstrual bleeding in research and clinical practice: a systematic literature review. BMC Womens Health.

